# The Dry Eye Disease Activity Log Study

**DOI:** 10.1100/2012/589875

**Published:** 2012-10-24

**Authors:** Jayant V. Iyer, Sze-Yee Lee, Louis Tong

**Affiliations:** ^1^Ocular Surface Research group, Singapore Eye Research Institute, Singapore 168751; ^2^Corneal and External Eye Services, Singapore National Eye Centre, Singapore 168751; ^3^Office of Clinical Science, Duke-NUS Graduate Medical School, Singapore 169857; ^4^Department of Ophthalmology, Yong Loo Lin School of Medicine, National University of Singapore, Singapore 119228

## Abstract

Prolonged visually stressful activities aggravate dry eye disease (DED). The duration spent on such activities and their relationship with DED clinical features were investigated. Patients completed an activity log as they performed their usual activities over 1 typical rest day and 1 typical work day. The log included time spent in an air-conditioned environment, windy environment, driving, watching television, computer use, reading, watching a movie in the theatre, and wearing contact lens. Average daily activity hours were calculated and correlated with clinical features of DED. Thirty-five logs were returned. Positive correlation was found between watching television and episodic blurred vision (*P* < 0.01). Computer use was negatively correlated with episodic blur vision, burning sensation, and gritty sensation (*P* < 0.05). Negative correlation was found between time spent in windy environments, driving, reading, and certain DED symptoms (*P* < 0.05). Reading correlated positively with severity of corneal fluorescein staining and reduced Schirmer's values (*P* < 0.03). The use of air conditioning correlated negatively with episodic blur vision but positively with visual blurring that improves with lubricants (*P* = 0.02). This study is the first to evaluate the relationship between time spent on DED-aggravating activities and DED clinical features. Negative correlations between certain activities and DED symptoms suggest an unconscious modification of lifestyle to alleviate symptoms.

## 1. Introduction

Dry eye disease (DED) or keratoconjunctivitis sicca is a common and often overlooked cause of distress in patients [1]. DED has been defined by the International Dry Eye Workshop [2] as a “multifactorial disease of tears and ocular surface that results in symptoms of discomfort, visual disturbance, and tear film instability with potential damage to ocular surface. It is accompanied by increased osmolality of tear film and inflammation of ocular surface.” 

DED has been found to dramatically decrease quality of life (QoL) [3–5], and it imposes a significant economic burden [6, 7]. DED patients may also experience more anxiety and depression, which is correlated to the severity of their symptoms [8]. Symptoms [9] include ocular burning, stinging, grittiness, foreign body sensation, tearing, ocular fatigue, and dryness. It may be episodic or chronic. 

Known risk factors for DED include increasing age [10], female sex [11], hormonal changes [12, 13], (e.g., post-menopausal) [14], eyelid disease [1], refractive surgery [15], autoimmune disease [16], and smoking [17]. Environmental risk factors for DED include contact lens use [1], low humidity [18] (e.g., air-conditioned environment), exposure to sun, dust and wind [19], medications [9]—topical eye-drops with preservatives and systemic medications like antihistamines and antidepressants—and extended visual tasking [20, 21] computer use, television use, prolonged reading, and driving.

Traditionally, practitioners utilize one-point-of-time tests such as Schirmer's test and tear breakup time (TBUT) in grading and management of patients with DED [22]. Overreliance on such tests may lead to incorrect diagnosis and inappropriate treatment for patients with DED. In addition, results of DED diagnostic tests do not give clinicians an indication of the impact of the disease on patients' QoL due to the poor concordance between clinical signs and patients' symptoms [23–25]. On the other hand, symptom assessment is very subjective and may not accurately reflect the effect of the condition on patients' lives.

Given the significant role played by extended visual tasking and environmental risk factors in DED exacerbation [18], behavior and activity modification may benefit the symptomatic DED patient. However, the clinician does not have a good measure of the extent of environmentally induced ocular surface stress encountered by DED patients. We hypothesize that a continuous record of the time spent in patients' selected daily activities and the waking hours may be a measure of the amount of ocular surface stress.

The dry eye disease activity log (DEDAL) study was performed with the following primary aims:

1. To report the “activity load”—mean amount of time spent per day on DED-aggravating activities—of DED patients from a tertiary care ophthalmic clinic.
2. To analyze any correlation between the “activity load” and the subjects' subjective symptoms or the traditionally utilized DED tests: Schirmer's test, TBUT, and corneal fluorescein staining.

## 2. Materials and Method

Subjects were DED patients consecutively recruited from the Singapore National Eye Centre Dry Eye Clinic. Written informed consent was obtained from each patient. This study conforms to the Declaration of Helsinki and was approved by the Institutional Review Board of the Singapore Eye Research Institute. Eligibility criteria included any patients above 21 years old referred to this clinic for management of dry eye and ability to understand the instructions of this study. There were no uniform criteria practiced in this center for referral as individual physicians determine whether the symptoms and signs of dry eye were significant enough to warrant referral.

Each subject was given a questionnaire and an activity log to complete and underwent a DED-specific ocular assessment performed by a single ophthalmologist. The questionnaire assessed subjects' medical, ophthalmic and medication history, and subjective symptoms relevant to dry eye. Patients were asked how often they were affected by the following symptoms in the previous month: light sensitivity, gritty ocular sensation, burning ocular sensation, episodic blurred vision, fluctuating vision with blinking, tearing, pain on waking in the morning, and visual improvement induced by artificial tears. The frequency of experiencing symptoms was graded as 0 (none), 1 (less than once a week), 2 (once a week), 3 (more than once a week but not daily), or 4 (at least once a day).

Ophthalmic assessment was performed by a single clinician (LT) to avoid interobserver discrepancies. Assessment included Schirmer's test I (without anesthesia), tear breakup time (TBUT), and corneal fluorescein dye staining. For Schirmer's test I, a 35 mm × 5 mm size filter paper strip (Sno strips, Bausch, and Lomb, NY) was used to measure the amount of tears produced over 5 minutes. The strip was placed in the inferior fornix at the junction of the middle and lateral thirds of the lower eyelid. The patient was instructed to keep their eyes closed during the course of the test. The level of tears on the strip was then read off and recorded in millimeters.

For the evaluation of TBUT and corneal fluorescein staining, a slit lamp biomicroscope at 10x objective was used with a broad beam focusing on the surface of the cornea, with maximal intensity of light illumination. Sodium fluorescein was instilled with a fluorescein strip (Fluorets, Bernell, USA) moistened in sodium chloride without measuring its volume and the strip shaken dry before use. 

After the instillation of fluorescein, the patient was then asked to blink three times and then look straight ahead without blinking. Without holding the eyelid, the tear film was observed under cobalt-blue filtered light of the slit lamp biomicroscope. The time that elapsed between the last blink and appearance of the first break in the tear film was recorded (a break is seen as a dark spot in a sea of green) as the TBUT. 

Corneal fluorescein staining was graded according to the Baylor staining scheme [26]. The cornea was divided into 5 zones (superior, inferior, nasal, temporal and central). The number of spots in each zone determined the grade of corneal fluorescein staining based on a 5-point scale: grade 0 = no staining, grade 1 = 1 to 5 spots, grade 2 = 6 to 15 spots, grade 3 = 16 to 40 spots and grade 4 = >40 spots. One point was added to the grade of the zone if there was confluent staining and a further point if filaments were observed in the zone.

To determine the severity of the DED conditions in our study population, the Delphi Consensus Dysfunctional Tear Syndrome (DTS) score [27] was generated for each patient based on the severity of symptoms and clinical signs (DTS 1 = mild DED and DTS 4 = severe DED).

**Figure 1:**
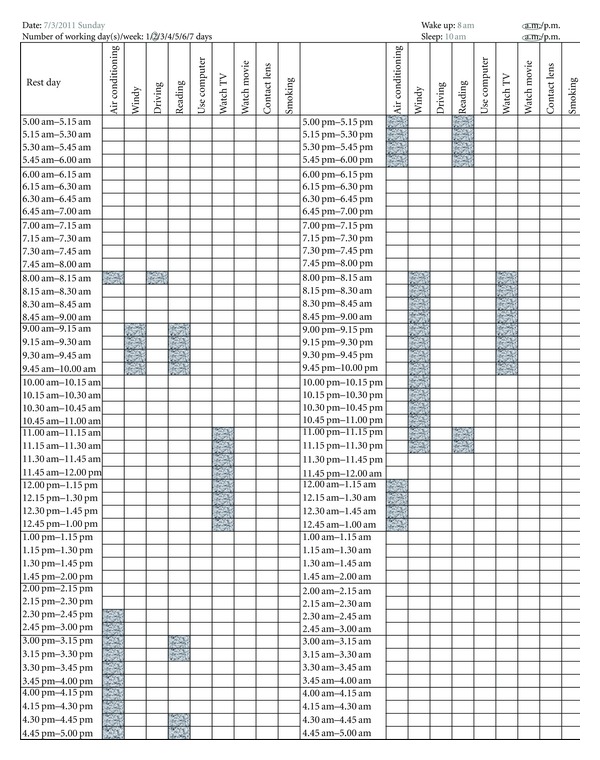
An example of an activity log as filled in by a patient.

On leaving the clinic, each patient was given an activity recording form and told to complete the form as they performed their usual activities (Figure 1) like a diary. In blocks of 15 minutes, time spent performing known DED-aggravating activities [18] was recorded in the log. Items assessed included time spent in air-conditioned environment, in windy environment, driving, reading, computer use, watching television, smoking, contact lens use, and watching movies in the theater. The last three items were initially included in the protocol but were removed later as very few participants used contact lens (*n* = 1), smoked cigarettes (*n* = 1), or spent time in the theatres (*n* = 3) in the study duration. Thus, further analyses of the activity load of these activities were not performed. Patients were instructed to return the completed form to the investigators by mail within 1 month.

Marking more than one column of the chart in any particular unit of time was permissible (Figure 1), when more than one item was satisfied concurrently. For example, if the subject was driving in an air-conditioned car, both the columns for “driving” and “air-conditioned environment” were marked in the activity log. A “windy environment” included an indoor situation exposed to a revolving fan or outdoor in a windy environment. 

The log was completed over 2 days, including 1 typical rest day and 1 typical work day. A variable was computed based on the weighted average of the number of rest days and work days each subject reported to have per week (usually 5 work days and 2 “rest” or weekend days).

To maintain independence of data points, only one eye per patient (selected to be the one with the worse clinical finding/subjective symptom) was included in the analysis. Results were analyzed using SPSS version 17.0. Significance was set at alpha = 0.05. Nonparametric correlation was calculated using the Spearman correlation coefficient (*r*).

## 3. Results

A total of 45 activity logs were distributed to 45 patients from the SNEC Dry Eye Clinic. Thirty-six logs were returned, of which one was improperly filled up and not included in the analysis (*n* = 35). Twenty-nine of the 35 subjects were female; 32 were Chinese, 1 was Indian, 1 was Burmese and 1 was Korean. Mean age was 57.  Table 1 summarizes the demographics of the subjects in the study. Subjects had moderate DED with an average DTS [27] (Delphi Consensus Dysfunctional Tear Syndrome, DTS 1 = mild DED and DTS 4 = severe DED) score of 2.66 ± 0.68 (mean ± SD).

The number of participants who engaged in spending time in air-conditioned environment, in windy environment, driving, reading, computer use or watching television was 29, 14, 11, 30, 17, and 34, respectively. 

**Figure 2:**
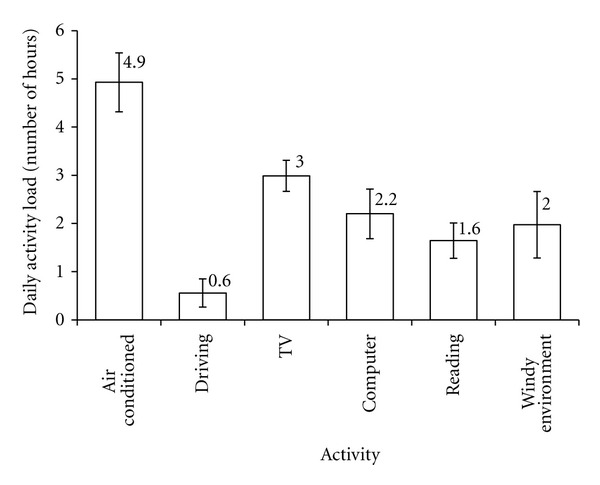
The average number of hours per day (of the 35 subjects) spent on each item.

The time spent for each item (Figure 2) and its correlation with perceived frequency of DED symptoms and clinical signs were calculated, as shown in Tables 2 and 3. 

Amount of computer use was negatively correlated with episodic blur vision (*P* = 0.048), burning sensation (*P* = 0.037), and gritty sensation (*P* = 0.034). Time spent reading and time spent in a windy environment were also negatively correlated with the frequency of experiencing burning sensation (*P* < 0.05). 

Patients who spent more time watching television experienced more episodic blur vision (*P* = 0.004). Time spent in an air-conditioned environment was negatively correlated with episodic blur vision but revealed a positive correlation with visual improvement with artificial tear usage (*P* < 0.05) (Table 2). 

Increased reading time was associated with more severe clinical signs of dry eye, particularly corneal fluorescein staining (*P* = 0.025) and Schirmer's test (*P* < 0.03) (Table 3). Finally, patients who spent more time driving (*P* = 0.046) and/or in a windy environment (*P* = 0.008) presented with more severe corneal staining in the superior region (Table 3).

## 4. Discussion

This study found the time spent on daily activities (reading, watching television, and computer use) and exposure to drying environments (air conditioning) to be associated with clinical symptoms or signs of DED. To the best of our knowledge, this was the first study undertaken to date examining the use of a daily recording log for charting the activities of patients with DED. The study revealed a few interesting findings. 

Patients who spent more time watching the television experienced more episodic blur vision. Watching television could be associated with a reduced blink rate leading to higher tear evaporation and tear film instability resulting in episodic blurred vision reported by the subjects.

On the other hand, exposure to windy environment, computer use, reading, and driving had negative correlations with frequency of certain dry eye symptoms. This could suggest activity modification due to DED; that is, symptomatic DED patients avoid windy environment or tend to drive for shorter durations. We note that driving time is very short for all the subjects in Singapore (Figure 2), and this limited range may affect the protocol to detect significant correlations.

Interestingly, exposure to an air-conditioned environment was correlated to increased frequency of blurring of vision reversible by lubricant use. Our finding may be explained either by greater frequency blurring of vision experienced in the dry environment or by an alteration in the nature of the blurring of vision in such environments. It has been demonstrated that the tear evaporation rate increases in low-humidity environments and subsequently disrupts tear film stability [18].

Subjects who spent more time reading were found to have more severe corneal fluorescein staining and poorer Schirmer's readings. As blink rate significantly reduces during reading [28], the interlink time interval would increase in patients who read extensively. This exposes the ocular surface to more stressful stimuli and may result in epitheliopathy as measured by staining. However, the patients with more severe dry eye may avoid prolonged reading, explaining the negative correlation between reading time and frequency of burning sensation. 

The study has a few limitations. Firstly, the study population is small and may not be representative of all DED patients; it largely comprised postmenopausal Chinese women with moderate DED (average DTS score 2.45). A larger group may be necessary to validate the significance of the activity recording. Secondly, the diary log was filled up over a period of only 2 days, which may not be accurately representative of the daily average “activity load” experienced by the subject. Thirdly, the study did not include tear osmolarity testing, which has been validated in other studies as to having the greatest diagnostic value among dry eye tests.

While tests such as Schirmer's and TBUT do have a role in objective assessment of DED, sole reliance of such tests may lead to an incomplete or inaccurate representation of the disease as the physician only assesses the patients in a very specific time in a confined artificial environment. It has been previously published that dry eye clinical signs were only seen in 57% of patients who had symptoms of dry eye [29].

Physicians are often busy and unable to conduct prolonged interviews concerning activities of daily living. Such history taking is also inaccurate because of recall bias. It may not be possible to assess the effect of activities by asking a single question since some patients may modify their behavior for one activity but not another. It may be difficult to explain why such patients alter their behavior for selective activities only. We suggest that such patients may be able to compensate for leisure type activities (e.g., reading a novel) but not for work-related ones (e.g., using the computer).

An activity log is a continuous monitoring tool, much like how a diet or exercise log is used in diabetes and weight management. Lifestyle modifications may be a very useful adjunct in the management of DED. The recording form may be used as a clinical tool to monitor the extent of behavioural adjustment and allow clinicians to recognize the impact of certain activities on the dry eye status of the patients.

In conclusion, we report the use of a diary type tool that records daily activities (reading, watching television, and computer use) and exposure to drying environments (air conditioning) in dry eye patients. We found that these are associated with specific clinical features of dry eye, and that some behavioral modification may have been present in these patients.

## Figures and Tables

**Table 1:** Patients' demographics and mean symptom frequency grade, clinical measure outcomes, and DTS grade.

	Female	Male	Total
Number of subjects (*n*)	29	6	35
Age (mean ± SD)	56.3 ± 11.4	59.0 ± 6.7	56.8 ± 10.7
Clinical measures (data from worse eye) (mean ± SD)			
TBUT (seconds)	2.3 ± 0.5	2.6 ± 1.0	2.5 ± 1.0
Schirmer's test I (mm)	10.8 ± 7.9	9.8 ± 8.3	10.0 ± 8.0
Corneal staining:			
(i) superior	1.3 ± 1.4	1.0 ± 1.0	1.0 ± 1.1
(ii) inferior	2.3 ± 1.0	1.7 ± 1.1	1.8 ± 1.3
(iii) nasal	2.0 ± 1.6	1.5 ± 1.2	1.4 ± 1.3
(iv) temporal	2.0 ± 1.3	1.3 ± 1.3	1.6 ± 1.3
(v) central	2.0 ± 1.3	1.4 ± 1.1	1.5 ± 1.2
DED symptom frequency (mean ± SD)			
Sensitivity to light	1.2 ± 1.8	1.5 ± 1.8	1.5 ± 1.8
Gritty sensation	1.8 ± 1.8	2.3 ± 1.7	2.3 ± 1.7
Burning sensation	0.4 ± 0.9	0.9 ± 1.4	0.9 ± 1.3
Episodic blur vision	2.0 ± 2.0	1.8 ± 1.7	1.9 ± 1.7
Fluctuating vision with blinking	0.4 ± 0.9	1.0 ± 1.5	0.9 ± 1.4
Tearing	1.0 ± 1.0	1.5 ± 1.7	1.4 ± 1.6
Pain on waking up in morning	0.2 ± 0.5	0.8 ± 1.4	0.7 ± 1.3
Need for artificial tear use	3.2 ± 1.8	2.0 ± 1.9	2.2 ± 1.9
DTS grade	2.7 ± 0.5	2.4 ± 0.8	2.7 ± 0.7

**Table 2:** Time spent for each item and correlations with DED symptoms. The table shows the Spearman's rho (*r*) and *P* values.

Activity	Correlation (Spearman's rho values) and significance (*P* values)					
	Sensitivity to light	Episodic blur vision	Burning sensation	Gritty sensation	Fluctuating vision with blinking	Blurring of vision relief by lubricants
Use of air conditioning	*r* = 0.265	*r* = −0.349	*r* = −0.146	*r* = −0.45	*r* = −0.52	*r* = 0.344
	*P* = 0.062	*P* = 0.020*	*P* = 0.201	*P* = 0.399	*P* = 0.383	*P* = 0.021^†^

Driving	*r* = − 0.07	*r* = − 0.355	*r* = − 0.049	*r* = − 0.26	*r* = − 0.247	*r* = 0.119
	*P* = 0.484	*P* = 0.018*	*P* = 0.390	*P* = 0.440	*P* = 0.076	*P* = 0.249

Watching television	*r* = 0.109	*r* = 0.447	*r* = 0.16	*r* = 0.118	*r* = 0.279	*r* = 0.130
	*P* = 0.267	*P* = 0.004^†^	*P* = 0.464	*P* = 0.249	*P* = 0.052	*P* = 0.229

Computer use	*r* = − 0.095	*r* = − 0.286	*r* = − 0.306	*r* = − 0.313	*r* = − 0.055	*r* = − 0.255
	*P* = 0.294	*P* = 0.048*	*P* = 0.037*	*P* = 0.034*	*P* = 0.378	*P* = 0.070

Reading	*r* = − 0.087	*r* = − 0.022	*r* = − 0.321	*r* = 0.017	*r* = 0.072	*r* = 0.19
	*P* = 0.309	*P* = 0.449	*P* = 0.030*	*P* = 0.461	*P* = 0.340	*P* = 0.137

Windy environment	*r* = − 0.089	*r* = − 0.226	*r* = − 0.411	*r* = 0.06	*r* = 0.37	*r* = − 0.002
	*P* = 0.306	*P* = 0.096	*P* = 0.007*	*P* = 0.366	*P* = 0.417	*P* = 0.495

*P < 0.05, negative correlation.

^†^ *P* < 0.05, positive correlation.

**Table 3:** And their relations with DED clinical signs represented with the Spearman's rho (*r*) and *P* values.

Activity	Correlation (Spearman's rho values) and significance (*P* values)						
	Corneal staining					Schirmer's test I	TBUT
	Superior	Inferior	Nasal	Temporal	Central
Use of air conditioning	*r* = 0.253	*r* = −0.217	*r* = −0.169	*r* = 0.119	*r* = 0.197	*r* = −0.151	*r* = 0.207
	*P* = 0.071	*P* = 0.105	*P* = 0.165	*P* = 0.248	*P* = 0.128	*P* = 0.193	*P* = 0.116

Driving	*r* = 0.289	*r* = −0.193	*r* = −0.021	*r* = 0.160	*r* = 0.174	*r* = −0.222	*r* = 0.122
	*P* = 0.046^†^	*P* = 0.134	*P* = 0.452	*P* = 0.179	*P* = 0.158	*P* = 0.100	*P* = 0.243

Watching television	*r* = −0.164	*r* = −0.176	*r* = −0.273	*r* = −0.176	*r* = −0.198	*r* = 0.048	*r* = 0.037
	*P* = 0.173	*P* = 0.155	*P* = 0.057	*P* = 0.156	*P* = 0.127	*P* = 0.393	*P* = 0.415

Computer use	*r* = 0.164	*r* = 0.002	*r* = 0.008	*r* = −0.017	*r* = −0.013	*r* = −0.234	*r* = 0.013
	*P* = 0.174	*P* = 0.496	*P* = 0.482	*P* = 0.460	*P* = 0.471	*P* = 0.088	*P* = 0.470

Reading	*r* = 0.335	*r* = −0.019	*r* = −0.027	*r* = 0.358	*r* = 0.320	*r* = −0.461	*r* = −0.096
	*P* = 0.025^†^	*P* = 0.456	*P* = 0.439	*P* = 0.017^†^	*P* = 0.030^†^	*P* = 0.005*	*P* = 0.292

Windy environment	*r* = 0.406	*r* = 0.080	*r* = 0.159	*r* = 0.216	*r* = 0.188	*r* = −0.094	*r* = 0.150
	*P* = 0.008^†^	*P* = 0.325	*P* = 0.181	*P* = 0.106	*P* = 0.140	*P* = 0.295	*P* = 0.195

*P < 0.05, negative correlation.

^†^ *P* < 0.05, positive correlation.
